# Induced Pluripotent Stem Cells in Congenital Long QT Syndrome: Research Progress and Clinical Applications

**DOI:** 10.31083/RCM28251

**Published:** 2025-04-22

**Authors:** Qing Li, Yi-Fei Wang, Bin Wang, Ting-Ting Lv, Ping Zhang

**Affiliations:** ^1^School of Clinical Medicine, Tsinghua University, 100084 Beijing, China; ^2^Department of Cardiology, Beijing Tsinghua Changgung Hospital, School of Clinical Medicine, Tsinghua University, 102218 Beijing, China

**Keywords:** congenital long QT syndrome, induced pluripotent stem cells, precision medicine

## Abstract

Congenital long QT syndrome (LQTS) is a potentially life-threatening hereditary arrhythmia characterized by a prolonged QT interval on electrocardiogram (ECG) due to delayed ventricular repolarization. This condition predisposes individuals to severe arrhythmic events, including ventricular tachycardia and sudden cardiac death. Traditional approaches to LQTS research and treatment are limited by an incomplete understanding of its gene-specific pathophysiology, variable clinical presentation, and the challenges associated with developing effective, personalized therapies. Recent advances in human induced pluripotent stem cell (iPSC) technology have opened new avenues for elucidating LQTS mechanisms and testing therapeutic strategies. By generating cardiomyocytes from patient-specific iPSCs (iPSC-CMs), it is now possible to recreate the patient’s genetic context and study LQTS in a controlled environment. This comprehensive review describes how iPSC technology deepens our understanding of LQTS and accelerates the development of tailored treatments, as well as ongoing challenges such as incomplete cell maturation and cellular heterogeneity.

## 1. Introduction

Congenital long QT syndrome (LQTS) is a rare hereditary ion channelopathy characterized by a prolonged QT interval and abnormal T wave morphologies on electrocardiogram (ECG). The prolongation of repolarization increases the risk of torsade de pointes (Tdp) ventricular tachycardia , which can result in syncope, cardiac arrest, and sudden cardiac death (SCD) [1, 2]. The estimated incidence of LQTS is approximately 1 in 2000 individuals [3], with a slight predominance in females. It accounts for 5% to 10% of SCD cases among young individuals [4]. The electrophysiological abnormalities are caused by gene mutations that disrupt the function of ion channels involved in action potential dynamics. These mainly include loss of function in the slow delayed rectifier potassium current (*I*_Ks_) and the rapid delayed rectifier potassium current (*I*_Kr_), and gain of function in the late sodium current (*I*_Na,L_) and the L-type calcium current (*I*_Ca,L_).

In terms of diagnosis, mutations in at least 17 genes have been associated with LQTS [5]. The Clinical Genome Resource (ClinGen) has recently reclassified these genes based on gene- and disease-specific criteria [6] (Table 1, Ref. [7, 8, 9, 10, 11, 12, 13, 14, 15, 16, 17, 18, 19, 20, 21, 22]). Seven genes were classified as definitive or strong evidence for causality. Among them, *KCNQ1*, potassium voltage-gated channel subfamily H member 2 (*KCNH2*), and sodium voltage-gated channel alpha subunit 5 (*SCN5A*) account for approximately 75% of cases. However, 25% of LQTS remains unexplained by known genes, suggesting the presence of undiscovered pathogenic genes [23, 24, 25]. LQTS shows incomplete penetrance, ranging from corrected QT interval (QTc) prolongation detectable as early as *in utero*, to lifelong asymptomatic carriers of pathogenic mutations. Moreover, family members carrying the same mutation may show variability in phenotype, with some experiencing sudden death while others remain asymptomatic throughout their lives. LQTS also exhibits significant genotypic heterogeneity, with distinct genetic subtypes that differ in age of onset, gender-specific risks, arrhythmogenic triggers, and therapeutic responses. Due to the lack of specific clinical manifestations, most LQTS cases cannot be classified based on clinical presentation or auxiliary examination alone (Schwartz score). Since precision treatment requires an accurate diagnosis, further interpretation of high-throughput sequencing results is needed to reveal genetic variation effects and to identify additional modifier factors.

**Table 1.** **Classification of genetic evidence by the Clinical Genome Resource (ClinGen) for genes previously associated with LQTS subtypes**.

Gene (Genotype)		Chromosomal Location	Protein	Functional effect	ClinGen evidence	Frequency	Reference
Long QT syndrome (Major)							
	*KCNQ1* (LQTS1)	11p15.5-p15.4	Potassium voltage-gated channel subfamily Q member 1	Reduced *I*_Ks_	Definitive	30–35%	Giudicessi *et al*. 2013 [7]
	*KCNH2* (LQTS2)	7q36.1	Potassium voltage-gated channel subfamily H member 2	Reduced *I*_Kr_	Definitive	25–30%	Curran *et al*. 1995 [8]
	*SCN5A* (LQTS3)	3p22.2	Sodium voltage-gated channel alpha subunit 5	Increased *I*_Na,L_	Definitive	5–10%	Wang *et al*. 1995 [9]
Long QT syndrome (Minor)							
	*CALM1*	14q32.11	Calmodulin-1	Abnormal Ca^2+^ signaling	Definitive*	<1%	Crotti *et al*. 2013 [10]
	*CALM2*	2p21	Calmodulin-2	Abnormal Ca^2+^ signaling	Definitive*	<1%	Crotti *et al*. 2013 [10]
	*CALM3*	19q13.32	Calmodulin-3	Abnormal Ca^2+^ signalling	Definitive*	<1%	Chaix *et al*. 2016 [11]
	*TRDN*	6q22.31	Triadin	N/A	Strong	Very rare	Altmann *et al*. 2015 [12]
	*CACNA1C*	12p13.33	Calcium voltage-gated channel subunit alpha1 C	Increased *I*_Ca,L_	Moderate/Definitive†	Very rare	Boczek *et al*. 2013 [13]
	*CAV3*	3p25.3	Caveolin-3	Increased *I*_Na_	Limited	<1%	Vatta *et al*. 2006 [14]
	*KCNE1*	21q22.12	Potassium voltage-gated channel subfamily E regulatory subunit 1	Reduced *I*_Ks_	Limited	<1%	Splawski *et al*. 1997 [15]
	*KCNJ2*	17q24.3	Potassium voltage-gated channel subfamily J member 2	Reduced *I*_K1_	Limited/Definitive#	<1%	Plaster *et al*. 2001 [16]
	*AKAP9*	7q21.2	A-kinase anchor protein 9	Reduced *I*_Ks_	Disputed	<1%	Chen *et al*. 2007 [17]
	*ANK2*	4q25-q26	Ankyrin-2	Abnormal ion channel localization	Disputed	<1%	Ichikawa *et al*. 2016 [18]
	*KCNE2*	21q22.11	Potassium voltage-gated channel subfamily E regulatory subunit 2	Reduced *I*_Kr_	Disputed/Definitive‡	Very rare	Abbott *et al*. 1999 [19]
	*KCNJ5*	11q24.3	Potassium voltage-gated channel subfamily J member 5	Reduced *I*_K,ACh_	Disputed	<1%	Yang *et al*. 2010 [20]
	*SCN4B*	11q23.3	Sodium voltage-gated channel beta subunit 4	Increased *I*_Na_	Disputed	<1%	Medeiros-Domingo *et al*. 2007 [21]
	*SNTA1*	20q11.21	Syntrophin alpha 1	Increased *I*_Na_	Disputed	<1%	Ueda *et al*. 2008 [22]

Notes: * Definitive (recurrent infantile cardiac arrest syndrome); † Definitive (Timothy syndrome); # Definitive (acquired LQTS); ‡ Definitive (Andersen-Tawil syndrome). LQTS, long QT syndrome; *I*_Ks_, slow delayed rectifier potassium current; *I*_Kr_, rapid delayed rectifier potassium current; *I*_Na,L_, late sodium current; *I*_Ca,L_, L-type calcium current; *I*_K,ACh_, acetylcholine-activated potassium current; *I*_K1_, inward rectifier potassium current; *I*_Na_, sodium current.

Comprehensive treatment of LQTS emphasizes β-blocker therapy (particularly nadolol and propranolol) as the cornerstone of management [26]. This is supplemented by sodium channel blockers (e.g., Mexiletine) for long QT syndrome type 3 (LQT3) [27], as well as more invasive interventions such as left cardiac sympathetic denervation (LCSD) [28] and implantable cardioverter-defibrillators (ICDs) for high-risk or treatment-resistant patients [29]. However, several challenges remain, such as elucidating the mechanisms of drug resistance, reducing drug side-effects to improve patient adherence, evaluating gene-specific novel drug development, and assessing biological therapy outcomes. In addition, the safety of drugs causing QTc prolongation must still be evaluated.

Traditional research models, including *in vivo*, *ex vivo*, and *in vitro* systems, have played an important role in elucidating gene-phenotype relationships and drug testing in LQTS [4, 30]. However, these models have significant limitations. Animal models have species-specific cardiac electrophysiological differences (e.g., mice lack specific *I*_Kr_, *I*_Ks_), making it difficult to accurately simulate human LQTS phenotypes. Cell models (e.g., human embryonic kidney 293 cells (HEK293)) often lack the intricate regulatory mechanisms present in cardiomyocytes. Moreover, obtaining primary human cardiomyocytes and maintaining them *in vitro* is challenging, which limits their use in disease modeling. Takahashi and Yamanaka [31] first reported induced pluripotent stem cells (iPSCs) in 2006, which have since become a transformative tool in LQTS research. By expressing specific transcription factors (e.g., Oct3/4, Sox2, c-Myc, Klf4), somatic cells (e.g., skin fibroblasts, peripheral blood mononuclear cells) can be reprogrammed into a pluripotent state. iPSCs are capable of differentiating into various cardiac cell types, including cardiomyocytes (iPSC-CMs), cardiac fibroblasts, endothelial cells (ECs), and smooth muscle cells (SMCs) [32]. This pluripotency provides a unique research platform for disease modeling, mechanistic studies, drug testing, and therapeutic development [33].

Patient-derived iPSC-CMs, or the introduction of mutations into healthy individual-derived iPSC-CMs through genome editing, offer several specific advantages compared to traditional models. These cells retain patient-specific genetic backgrounds, maintain the intrinsic cellular environment of human cardiomyocytes, and exhibit comparable ion current densities to native cells, thus accurately reproducing the action potential prolongation and arrhythmogenic susceptibility observed in LQTS patients [34, 35]. Moreover, the integration of gene editing technologies (e.g., CRISPR/Cas9) with iPSC-CMs enables the creation of isogenic cell lines, which eliminates confounding genetic factors and allows precise investigation of mutation-specific effects [36]. This advanced cellular model facilitates high-throughput drug screening and the development of targeted therapeutic strategies, thereby advancing precision medicine for LQTS patients.

Although significant progress has been made, challenges remain in the use of iPSC-CMs for LQTS research, including incomplete maturation of cardiomyocytes and the heterogeneity of differentiated cell types. Current iPSC-CMs often exhibit immature structural and functional phenotypes compared to adult cardiomyocytes, which may affect the interpretation of drug responses and the prediction of clinical outcomes. Additionally, differentiated cardiomyocytes are heterogeneous and comprise various subtypes, such as ventricular-like cells, atrial-like cells, and pacemaker cells, thus complicating the establishment of specific disease models. Advances in chemical, physical, and spatial techniques, as well as the development of engineered heart tissues (EHTs) or organoids, have improved cardiomyocyte maturation and subtype specificity. However, the development of unified evaluation standards are needed to improve the clinical predictive ability of iPSC-CMs. This review aims to deepen our understanding of LQTS and provide a comprehensive reference for the application of iPSCs in future research on precision medicine.

## 2. Recapitulation of Patient Clinical Phenotypes

IPSC-CMs have become essential tools for modeling LQTS by faithfully replicating patient-specific phenotypes. These models retain patient-specific genetic mutations and altered ion channel functions (e.g., reduced *I*_Ks_/*I*_Kr_ or enhanced *I*_Na,L_), effectively capturing clinical features of LQTS such as prolonged action potential duration/field potential duration (APD/FPD), arrhythmia susceptibility, and abnormal responses to beta-adrenergic stimulation, reflecting the complex pathophysiology of LQTS. An early study by Moretti *et al*. (2010) [37] reported an LQT1 model with the *KCNQ1*-p. R190Q mutation, replicating prolonged APD, reduced *I*_Ks_ current, and heightened catecholamine sensitivity. Building on this, Itzhaki *et al*. (2011) [38] created an LQT2 model with the *KCNH2*-p. A614V mutation, demonstrating reduced *I*_Kr_, early afterdepolarizations (EADs), and ventricular arrhythmias. Davis *et al*. (2012) [39] further extended these studies by developing an overlap syndrome model (LQT3 overlap) with the *SCN5A*-p. 1795insD mutation that showed prolonged APD and increased persistent *I*_Na,L_. Ma *et al*. (2013) [40] constructed an LQT3 model with the *SCN5A*-p.V1763M, successfully replicating the significantly prolonged APD and increased *l*_Na,L_ that are characteristic of LQT3.

Advances in iPSC technology have significantly improved model construction and phenotyping. Efficient differentiation protocols now yield more mature iPSC-CMs that resemble adult cardiomyocytes. Gene editing techniques, such as CRISPR/Cas9, enable the precise introduction and correction of mutations, while isogenic controls allow direct comparison with wild-type cells. A variety of electrophysiological techniques have been widely applied in the study of iPSC-CMs. These include Patch Clamp technology, which can detect cell membrane potential (e.g., resting membrane potential, action potentials), ion currents (e.g., *I*_Ks_, *I*_Kr_, *I*_Na_, *I*_Ca_), and current-voltage relationships (I-V curves). Patch Clamp also allows the analysis of ion channel dynamics and cell signal transduction processes. In particular, Automated Patch Clamp (APC) systems can significantly increase the throughput of single-cell iPSC-CMs electrophysiological analyses. Multi-Electrode Array (MEA) technology can record the electrical activity of cell populations (field potentials), monitor intercellular conduction (e.g., propagation patterns and conduction velocity), while observing the effects of drugs on electrical activity. Optical imaging using voltage-sensitive dyes and calcium indicators enables the measurement of membrane potential changes (e.g., action potentials and electrical signal propagation) and intracellular calcium dynamics (e.g., calcium transients and excitation-contraction coupling) in cardiac tissues. Such technological advances have allowed iPSC-CMs to model various LQTS subtypes, including LQTS types 1~3 and multisystem LQTS gene types (Table 2, Ref. [15, 37, 38, 39, 40, 41, 42, 43, 44, 45]). Recent research has increasingly focused on calcium abnormalities, including impaired calcium release, calcium-dependent inactivation, and altered excitation-contraction coupling. These have been identified as core features in many LQTS subtypes, such as calmodulinopathy, triadin knockout syndrome (TKOS), Jervell and Lange-Nielsen syndrome (JLNS), and Andersen-Tawil syndrome (ATS). Wren *et al*. (2019) [41] created several calmodulin mutation models (*CALM1*-p.E141V, *CALM3*-p.E141K, *CALM3*-p.D130G) and reported prolonged APD, calcium dysregulation, and mosaicism, highlighting the role of calmodulin in arrhythmogenesis. Similarly, Clemens *et al*. (2023) [43] used iPSC-CMs to replicate TKOS with triadin (*TRDN*)-c.22+29A>G and *TRDN*-c.484+1189G>A, demonstrating impaired excitation-contraction coupling. Kuroda *et al*. (2017) [44] employed iPSC-CMs to study ATS with potassium voltage-gated channel subfamily J member 2 (*KCNJ2*)-p. R218W, *KCNJ2*-p.R67W and *KCNJ2*-p.R218Q, finding abnormal Ca^2+^ release and ventricular tachycardia. Zhang *et al*. (2014) [45] modeled JLNS with the potassium voltage-gated channel subfamily Q member 1 (*KCNQ1*)-p.G160fs+138X and *KCNQ1*-p.R594Q homozygous mutations, thereby replicating prolonged APD and enhanced catecholamine sensitivity. Wang *et al*. (2022) [46] constructed a dual mutation model (*KCNQ1*-p.A219T and *TRPM4*-p.479C>T), revealing complex genetic interactions. These studies provide key insights into the pathophysiology of LQTS and its complex genetic background.

**Table 2.** **Summary of studies using iPSC-CMs to recapitulate clinical phenotypes of LQTS patients**.

LQTS subtype	Gene mutation	Cell phenotype	Experimental approaches	Reference
*KCNQ1 *(LQTS1)	p.R190Q	Reduced *I*_Ks_ current Prolonged APD	Patch clamp	Moretti *et al*., 2010 [37]
*KCNH2* (LQTS2)	p.A614V	Reduced* I*_Kr_ current	Patch clamp	Itzhaki *et al*., 2011 [38]
		Prolonged APD, arrhythmias		
*SCN5A* (LQTS3)	p.V1763M	Increased *I*_Na,L_ current	Patch clamp	Ma *et al*., 2013 [40]
		Prolonged APD, arrhythmias		
*SCN5A* overlap	p.1795insD	Persistent *I*_Na,L_ current Prolonged APD	Patch clamp	Davis *et al*., 2012 [39]
*CALM1*	p.E141V	Impaired CDI of L-type calcium current, prolonged APD, arrhythmias	Patch clamp; Ca^2+^ imaging	Wren *et al*., 2019 [41]
*CALM2*	p.N98S	Impaired CDI of L-type calcium current, prolonged APD, arrhythmias	Patch clamp; Ca^2+^ imaging	Limpitikul *et al*., 2017 [42]
*CALM3*	p. E141K	Prolonged APD interval, Impaired Ca^2+^ binding	Patch clamp; Ca^2+^ imaging	Wren *et al*., 2019 [41]
*TM*	*CACNA1C*	Prolonged APD, Abnormal calcium handling	Patch clamp; Ca^2+^ imaging	Splawski *et al*., 1997 [15]
	p.G406R			
*TKOS*	*TRDN*	Prolonged APD, complete loss of Triadin protein	Patch clamp; Ca^2+^ imaging; MEA	Clemens *et al*., 2023 [43]
	p.D18fs*13			
*ATS*	*KCNJ2*	Abnormal calcium handling	Patch clamp; Ca^2+^ imaging; MEA	Kuroda *et al*., 2017 [44]
	p.R218W			
	p.R67W			
	p.R218Q			
*JLNS*	*KCNE1*	APD/FPD prolongation with homozygous mutations	Patch clamp; MEA	Zhang *et al*., 2014 [45]
	p.G160fs+138X, p.R594Q			

APD, Action Potential Duration; FPD, Field Potential Duration; iPSC-CMs, Patient-specific induced pluripotent stem cell-derived cardiomyocytes; MEA, Multi-Electrode Array; CDI, Calcium-Dependent Inactivation.

## 3. Functional Validation of Mutations and Mechanistic Research

### 3.1 Validation of Variants of Uncertain Significance

The validation of Variants of Uncertain Significance (VUS) in LQTS using iPSC-CMs is a critical step towards gaining a better understanding of their pathogenicity. CRISPR/Cas9 gene editing is used to insert the variant into iPSCs derived from healthy individuals in order to determine whether it causes disease symptoms. Alternatively, gene editing can be used to correct the variant in patient-derived iPSCs to determine whether this reverses the disease phenotype. This approach allows investigation of the sufficiency and necessity of mutations for causing disease, while maintaining the same genetic background. For example, Garg *et al*. (2018) [47] found that patient-derived iPSC-CMs carrying the *KCNH2*-p.T983I variant exhibited significantly prolonged APD. Although 12.9% of the cells displayed arrhythmic activity such as EADs, the iPSC model demonstrated that even mild arrhythmias can be captured in LQTS studies. Treatment with a human ether-a-go-go-related gene (hERG) channel activator normalized APD, confirming the accuracy of the iPSC-CMs in reflecting patient responses. After introducing the same *KCNH2*-p.T983I variant into healthy control cells, the researchers observed an even more severe LQTS phenotype, further supporting the role of this mutation in LQTS. Conversely, correcting the variant in patient-derived cells restored normal phenotypes, including typical APD, and reduced arrhythmic activity. Interestingly, previous studies conducted using heterologous systems with gene editing may have led to incorrect conclusions regarding the pathogenicity of certain variants. For instance, the *KCNQ1*-p.A341V variant exhibited mild effects *in vitro*, but severe clinical symptoms [48], whereas the *KCNQ1*-p.Y111C variant showed severe loss of function *in vitro*, despite a low clinical risk of cardiac events [49]. These discrepancies can often be explained by differences in post-translational modifications or variations in the expression level of regulatory proteins in native human myocardium.

With the increasing use of next-generation sequencing (NGS), the number of identified gene variants linked to specific clinical phenotypes has grown exponentially, leading to an increase in uncharacterized VUS [50, 51]. The proportion of VUS varies across LQTS subtypes, ranging from 15–20% in LQT1, to over 30% in LQT3 due to the complexity of the *SCN5A *gene. For less common subtypes such as those involving the *CACNA1C* and *CALM* genes, VUS can account for up to 40–50% of the variants. By leveraging iPSC models and gene editing, researchers have been able to reclassify many VUS from uncertain to pathogenic or benign. For example, Chavali *et al*. (2019) [52] introduced the *CACNA1C*-p.N639T variant into a healthy iPSC line, resulting in significantly prolonged repolarization time and impaired calcium current inactivation, hence leading to its reclassification as likely pathogenic. Similarly, Yamamoto *et al*. (2017) [53] used patient-derived iPSC models to study the impact of the *CALM2*-p.N98S mutation on L-type calcium channel function, finding that it prolonged APD. Allele-specific silencing using CRISPR/Cas9 further demonstrated that removing the mutant allele could restore normal function, thereby providing key insights into the reclassification of VUS. iPSCs have also been used for functional validation of newly discovered LQTS genes. For example, Zhou *et al*. (2023) [54] combined genome sequencing and patient-specific sparagine-linked glycosylation 10B gene (*ALG10B*)-p.G6S iPSC-CMs. Compared to isogenic controls, these exhibited significantly prolonged APD and notable retention of hERG protein in the endoplasmic reticulum. Moreover, Lumacaftor rescue experiments systematically verified that *ALG10B*-p.G6S mutation leads to the LQTS phenotype by affecting hERG trafficking, suggesting that *ALG10B* may be a novel LQTS gene. These experiments demonstrate the importance of iPSC models used in combination with genome editing as a robust platform for understanding the functional impact of VUS and of new genes, thus improving the accuracy of LQTS diagnosis.

### 3.2 Evaluation of Modifier Genes

Modifier genes significantly influence the severity and clinical presentation of LQTS. While not directly causing LQTS, they modify how the disease manifests in individuals with pathogenic mutations. Modifier genes help to explain why patients with the same primary LQTS mutation can have different symptoms and outcomes. The identification of modifier genes typically involves three main approaches [55]: (1) Candidate gene approach. This targeted approach focuses on genes and genetic variants that are already known to be associated with QT interval and arrhythmias. For example, the *NOS1AP* gene is linked to QT interval prolongation and is considered to be a key modifier gene for LQTS [48]. (2) Genome-wide association studies (GWAS). This unbiased approach scans the entire genome for genetic variants linked to disease risk, thus helping to identify novel genetic markers or modifiers without relying on prior knowledge. (3) Family and founder population studies. The investigation of family members with the same pathogenic mutation but different clinical outcomes can reveal potential modifier genes.

iPSC-CMs provide a valuable platform for studying modifier genes. For example, Chai *et al*. (2018) [56] combined electrophysiological measurements of iPSC-CMs with whole-exome sequencing (WES) to identify two modifier genes associated with the LQT2 phenotype: *KCNK17 *and *REM2*. *KCNK17* encodes a two-pore potassium channel that may counteract the repolarization defects caused by *KCNH2 *mutations by increasing the potassium current, thereby providing a protective effect. *REM2* encodes a GTP-binding protein that regulates calcium channels. The *REM2*-p.G96A variant was found only in severe cases and was linked to increased *I*_Ca,L_. Correcting this variant with CRISPR/Cas9 successfully restored the normal phenotype in patient cells. In addition, Lee *et al*. (2021) [49] identified two single nucleotide variants (SNVs) of the *MTMR4* gene in asymptomatic LQTS iPSC-CMs that weakened Nedd4L activity and prevented excessive degradation of ion channels. After correcting these SNVs with CRISPR/Cas9, the ion channel degradation and electrophysiological characteristics of these cells were similar to those of symptomatic patients, thus confirming their protective role. It should be noted that each method for identifying modifier genes has its strengths and limitations, and they are often used in combination to enhance the accuracy of the findings.

### 3.3 Exploration of Pathological Mechanisms

The primary pathological mechanism of LQTS involves dysfunction of multiple ion channels, particularly potassium and sodium, and abnormal calcium handling which leads to abnormalities in cardiac action potentials [57, 58].

The *KCNQ1* gene encodes for the alpha subunit of a potassium channel that generates the *I*_Ks_ during cardiac repolarization. When *KCNQ1* is mutated (e.g., the *KCNQ1*-p.Y111C mutation [49]), the folding and transport functions of this channel are impaired and it is prevented from reaching the cell membrane. In patient-derived iPSC-CMs models, *KCNQ*1 mutations have been associated with reduced potassium ion flow, delayed repolarization, and QT interval prolongation. These abnormalities make cardiomyocytes more unstable during repolarization, increasing the risk of ventricular arrhythmias. Recent studies have highlighted the role of complex splicing events and protein degradation pathways in LQTS pathology. For example, Wuriyanghai *et al*. (2018) [59] reported that the *KCNQ1*-p.A344Aspl mutation led to aberrant splicing, potassium channel dysfunction, and increased EADs. These observations enhance our understanding of how splicing abnormalities can exacerbate LQTS. At the cellular level, the mutated *KCNQ1* protein was either misfolded or failed to properly localize to the cardiomyocyte membrane, causing a significant reduction in *I*_Ks_ current density. This reduction results in prolonged action potential repolarization, which directly manifests as QT interval prolongation on an ECG.

The *KCNH2* gene encodes a voltage-gated potassium channel that generates the *I*_Kr_ and mutations that cause incorrect folding or intracellular retention of the channel prevent its proper insertion into the cardiomyocyte membrane [60]. iPSC-CMs models have shown that hERG channel dysfunctions lead to reduced *I*_Kr_ currents, further impairing cardiac repolarization and prolonging the QT interval. Studies have also shown that *KCNQ1* mutations may indirectly affect *I*_Kr_ through enhanced degradation. For example, some *KCNQ1* mutations may increase the degradation of both KCNQ1 and hERG proteins through the upregulation of regulatory proteins such as Nedd4L, further weakening repolarization and worsening the LQTS phenotype. Additionally, research by Feng *et al*. (2021) [61] on the *KCNH2* mutation hERG1-p.H70R in iPSC-CMs models suggests these mutations lead to decreased *I*_Kr_ currents and accelerated deactivation in cardiomyocytes, directly contributing to prolonged APD and consistent with the LQTS phenotype. The main molecular mechanism involves a reduction in the complex glycosylation forms of hERG1a protein, while the expression of hERG1b remains unaffected. This imbalance of subunits weakens *I*_Kr_ currents, with abnormal folding and trafficking defects in hERG1a leading to endoplasmic reticulum-associated degradation that reduces the expression of functional channels. The complexity of this molecular mechanism was further revealed by analysis of mRNA splicing and post-translational regulation, emphasizing the impact of subunit ratios on channel function.

*SCN5A* gene mutations delay inactivation of the NaV1.5 sodium channel and increase *I*_Na,L_, causing persistent sodium influx. This prolongs APD and ventricular repolarization, contributing to inherited arrhythmias like LQT3 and Brugada syndrome. Kroncke *et al*. (2019) [62] found that iPSC-CMs with the p.R1193Q variant exhibited significant late sodium current, leading to prolonged action potentials and triggered activity in cardiomyocytes. These features could be reversed by the addition of phosphatidylinositol (3,4,5)-trisphosphate (PIP3), suggesting that the pathogenic mechanism of the p.R1193Q variant may involve PIP3-related regulatory pathways rather than mechanisms of direct channel inactivation. Additionally, Nieto-Marín *et al*. (2022) [63] used iPSC-CMs with *Tbx5* gene mutations (p. D111Y and p. F206L) to examine changes in *SCN5A* gene expression and the resulting functional effects on the cardiac sodium channel NaV1.5. These authors found the *Tbx5*-p.D111Y variant increased the expression of CaMKII and its anchoring proteins, leading to an increase in the *I*_Na,L_ of Nav1.5 and thus prolonging the action potential duration.

Calcium homeostasis also plays an important role in the pathophysiology of LQTS. Mutations in *CALM* impair calcium binding, resulting in abnormal calcium transients, prolonged APD, and an increased risk of arrhythmias such as EADs and delayed afterdepolarizations (DADs). Ledford *et al*. (2020) [64] showed that LQTS-related mutations such as *CALM*-p.D96V and *CALM*-p.D130G significantly reduce small-conductance Ca^2+^-activated K^+^ channel currents through a dominant-negative effect. These *CALM* mutations may therefore worsen LQTS pathology by impairing Ca^2+^-dependent repolarization currents. The *CALM*-p. D96V mutation led to a significant reduction in apamin-sensitive currents in iPSC-CMs, whereas the inhibitory effect of *CALM*-p.F90L was weaker, indicating that different *CALM* mutations may affect small-conductance Ca^2+^-activated K^+^ channel function through distinct mechanisms. The *CALM*-p.D96V mutation, in particular, severely affected channel function by impairing Ca^2+^ binding.

In summary, understanding the pathophysiology of LQTS involves exploration of how various ion channel dysfunctions and abnormal calcium handling produce the LQTS clinical phenotype. The resulting knowledge framework provides a scientific basis to support targeted therapeutic strategies and personalized management of this life-threatening disorder.

## 4. Therapeutic Strategies

### 4.1 Valuation Genotype-Specific Drug 

Genotype-specific treatments have shown significant progress for congenital LQTS. In particular, beta-blockers can effectively reduce cardiac events by over 95% for LQT1, 75% for LQT2, and 60% for LQT3. Propranolol, atenolol, and nadolol have shown efficacy, whereas metoprolol is less effective. Chockalingam *et al*. (2012) [26] suggested that in addition to their membrane stabilizing effect, propranolol and nadolol block peak *I*_Na_, and affect non-inactivating *I*_Na,L_, thus further contributing to APD shortening. Sodium channel blockers may also be required for symptomatic LQT3 patients. Ma *et al*. (2013) [40] reported that mexiletine reduces *I*_Na,L_, restores peak *I*_Na_, and improves electrophysiological properties. However, limitations remain with the current treatments. Despite advances in genetics, around 25% of LQTS cases lack an identified causative gene, thereby complicating precise risk assessment and targeted treatment. Furthermore, even within the same family, individuals with the same mutation can exhibit different disease severities, making risk assessment and treatment planning challenging. In severe cases, such as newborns with certain *SCN5A*, *CALM*, or *TRDN* mutations, current therapies often fail, highlighting the need for more effective drugs. Additionally, the side-effects of beta-blockers can impair patient adherence, particularly in those with no or mild symptoms, increasing the risk of arrhythmic events. Although effective for some LQTS types, sodium channel blockers can have off-target effects that add to the complexity of treatment. Mutation-specific therapies, such as lumacaftor, are only effective for specific cases and are not broadly applicable across all LQTS types. These challenges highlight the need to better understand the genetic complexity of LQTS, improve risk assessment, and develop more effective therapies that target the diverse presentations of this condition.

iPSCs hold significant value in the study of gene-specific drug therapies because they provide a patient-specific cellular environment that enables the evaluation of drug efficacy, ion channel specificity, off-target effects, and potential side-effects. Comollo *et al*. (2022) [65] demonstrated that propranolol effectively inhibited late sodium current (*I*_Na,L_) in LQT3 cardiomyocytes with LQT3-associated a three amino acid deletion (p.ΔKPQ, Δ=deletion, K=Lysine, P=Proline, Q=Glutamine) and p.E1784K mutations. The inhibitory effect was more pronounced for the latter mutation, and the action of propranolol on *I*_Na,L_ was stronger than its effect on the *I*_Kr_ channel. These observations indicate that propranolol may have minimal off-target effects and potentially fewer side-effects for the treatment of LQT3. Stutzman *et al*. (2023) [66] highlighted the importance of using iPSC-CMs for the functional characterization of new mutations such as *SCN5A*-p.F1760C, especially after the failure of conventional treatments. They found the p.F1760C mutation led to increased *I*_Na,L_ and a shift in channel inactivation, which traditional sodium channel blockers such as mexiletine could not manage effectively. However, phenytoin successfully reduced the prolonged APD in these cells, suggesting it may be an alternative therapy for LQT3 patients who are resistant to standard treatments. These examples demonstrate the utility of iPSC-CMs in understanding the impact of specific mutations and identifying targeted therapies, allowing for personalized treatment strategies in challenging cases. The success of phenytoin in restoring normal electrophysiological function in p.F1760C mutant cells suggests that repurposing anti-epileptic drugs could be an effective approach for treating variants that interfere with traditional drug binding. This case underscores the role of patient-specific cell models in bridging the gap between genetic diagnosis and effective therapy for channelopathies.

### 4.2 Drug Screening and Discovery

The comprehensive *in vitro* proarrhythmia assay (CiPA) is a new framework that integrates human-based computational models, *in vitro* assays, and iPSC-CMs. Due to their human origin and functional characteristics, iPSC-CMs are an effective platform for assessing drug-induced arrhythmias and other cardiac toxicities. Using multiparametric evaluations including electrophysiology, calcium signaling, cellular structure, and metabolic activity, in combination with high-content screening, iPSC-CMs are able to detect both arrhythmic and structural toxicities. When tested with drugs that are known to have arrhythmic risks, such as dofetilide and sotalol, iPSC-CMs accurately predicted the drugs’ potential to prolong APD and trigger arrhythmic activity, demonstrating their value in evaluating early toxicity to identify high-risk compounds. Therefore, iPSC-CMs serve as a powerful platform for testing drug efficacy against specific mutations and for uncovering potential anti-arrhythmic effects (Table 3, Ref. [40, 46, 67, 68, 69, 70, 71, 72, 73]). For example, Mehta *et al*. (2018) [72] used iPSC-CMs from LQT2 patients to evaluate the effect of Lumacaftor, a drug originally designed for cystic fibrosis. LQT2 is primarily caused by mutations in the *KCNH2* gene, resulting in reduced *I*_Kr_ current due to hERG potassium channel misfolding and defective trafficking to the cell membrane. The authors showed that Lumacaftor successfully corrected these defects in iPSC-CMs to restore the normal function of hERG channels. Further validation of these findings were provided by Schwartz *et al*. (2019) [74], who conducted a clinical trial using a combination of Lumacaftor and Ivacaftor (LUM+IVA) in two LQT2 patients. Consistent with *in vitro*results, both patients showed a significant reduction in QTc interval, albeit to a smaller degree compared to the cell studies. Although side-effects such as diarrhea were observed, the reduction in QTc interval during treatment, along with its subsequent rebound during drug washout, suggested a promising therapeutic effect. Another significant development was reported by Giannetti *et al*. (2023) [73], who explored the effects of serum/glucocorticoid-regulated kinase 1 (SGK1) inhibitors. They found that SGK1 inhibition consistently shortened APD in LQT2 models, but showed inconsistent outcomes in LQT1 models. This result suggests that SGK1 inhibitors have potential for genotype-specific therapeutic use, and reinforce the importance of genetic testing in guiding effective treatments. Overall, these advances illustrate how novel screening approaches and iPSC-CMs models are paving the way for more precise, personalized treatment of LQTS.

**Table 3.** **Overview of published drug discovery in iPSC-CM arrhythmia models**.

Drug		Mode of action category		Subtype	Gene mutation	Effect on phenotype	Reference
*I*_Kr_ activators or modulators							
	ICA-105574	Activates *I*_Kr_, restores Kv11.1 current	LQT2	*KCNH2*	p.T618S, p.N633S, p.A422T	Reduces FPD, risk of overcorrection	Perry *et al*., 2020 [67]
	NS1643	Activates *I*_Kr_	LQT2	*KCNH2*	p.A561T	Increased *I*_Kr_, shortened APD by 20%	Duncan *et al*., 2017 [68]
	PD118057	Activates *I*_Kr_	LQT2	*KCNH2*	p.F617L	Increased *I*_Kr_, shortened APD by 17.3%, reduced arrhythmic activity	Matsa *et al*., 2011 [69]
	LUF7346	Activates *I*_Kr_, type-1 hERG allosteric modulator	LQT2	*KCNH2*	p.N996I	Increased *I*_Kr_, shortened APD by 15–45%, reduced arrhythmic activity	Sala *et al*., 2016 [70]
Sodium channel blockers							
	Mexiletine	Sodium channel blocker	LQT3	*SCN5A*	p.V1763M	*I*_Na,L_ inhibition and shortened APD by 20%	Ma *et al*., 2013 [40]
	Mexiletine analogues	Sodium channel blocker	LQT3	*SCN5A*	p.P1473C p.A406L	*I*_Na,L_ inhibition and APD shortening at lower doses; analogues ‘MexA20’ and ‘MexA50’ more potent and selective, resulting in APD shortening and arrhythmia suppression	Cashman *et al*., 2021 [71]
Calcium channel blockers							
	Verapamil	Calcium channel blocker	LQT1	*KCNQ1/TRPM4*	p.G219E/p.T160M	Significantly shortened APD, reduced the risk of arrhythmias	Wang *et al*., 2022 [46]
PPAR-δ agonists							
	Telmisartan, GW0742	PPAR-δ agonists, reduce repolarization abnormalities	LQT2	*KCNH2*	p.A561T	Reduction in repolarization abnormalities, shortened APD by 19%, suppressed arrhythmic events	Duncan *et al*., 2017 [68]
ATP-sensitive potassium channel K_ATP activators							
	Nicorandil	Activates ATP-sensitive potassium channels K_ATP	LQT2	*KCNH2*	p.A561T	Shortened APD by up to 21%	Duncan *et al*., 2017 [68]
	Minoxidil	Activates ATP-sensitive potassium channels K_ATP	LQT2	*KCNH2*	p.A561T	Shortened APD by up to 17%, some significant reductions in triangularization	Duncan *et al*., 2017 [68]
Protein trafficking potentiators							
	Lumacaftor	Acts as a chaperone for hERG protein, correcting trafficking defects and stabilizing calcium handling	LQT2	*KCNH2*	p.A561V, p.IVS9-28A/G	Shortened FPD by 30–40%, improved hERG trafficking to the cell membrane and reduced calcium-handling irregularities	Mehta *et al*., 2018 [72]
Serum/Glucocortmpm-requlated kinase (SGK1) inhibitors (SGK1-I)							
	SGK1-Inh	Inhibits serum/glucocorticoid-regulated kinase 1	LQT1, LQT2	*KCNH2*	p.A561V, p.R594Q	Dose-dependent shortening of FPD and APD by 20–45% in LQT2, variable effects in LQT1 models, with significant FPD/APD shortening only at higher concentrations	Giannetti *et al*., 2023 [73]
				KCNQ1			

PPAR-δ, peroxisome proliferator-activated receptor; LQT1, long QT syndrome type 1; hERG, human ether-a-go-go-related gene; PD, pfizer discovery; LUF, leiden university; NS, NeuroSearch; ICA, icagen.

### 4.3 Biological Therapy

Biological therapies offer a promising new direction for treating LQTS by targeting the molecular root of the disorder. Suppression and replacement gene therapies have shown potential for addressing genetic mutations associated with LQTS. Dotzler *et al*. (2021) [75] reported that a novel suppression-replacement gene therapy (*KCNQ1*-SupRep) for LQT1 was an effective way to address mutant alleles while introducing functional ones. The suppression phase involved short hairpin RNA (shRNA) targeted at *KCNQ1* and which reduced the expression of both mutant and wild-type alleles to mitigate harmful gene effects. The replacement phase used an immune-compatible *KCNQ1* complementary DNA (cDNA) variant (shIMM) that was resistant to shRNA suppression and restored normal *KCNQ1* protein function. This combined approach successfully shortened APD in iPSC-CMs and normalized the pathological features in a transgenic rabbit model of LQT1.

Optogenetic therapies have also emerged as potential tools for precise control of cardiac electrophysiology. Gruber *et al*. (2021) [76] explored the use of light-sensitive ion channels to regulate APD in iPSC-CMs from LQTS patients. Their approach used channelrhodopsin-2 (ChR2), a cation channel activated by blue light, to trigger the influx of sodium and calcium ions and thus cause cellular depolarization. By applying light at specific AP phases, these authors achieved bidirectional APD modulation: activation during phase 3 (repolarization) extended APD, whereas activation during phase 2 (early repolarization) shortened it. They also used anion channelrhodopsin-2 (ACR2), a chloride-selective channel, to shorten APD by promoting hyperpolarization. These optogenetic interventions showed promise in restoring normal APD and lowering arrhythmia risk, indicating that optogenetics may be a potential regulatory strategy for cardiac arrhythmias.

Additionally, monoclonal antibody therapy targeting the *KCNQ1* potassium channel has shown potential for treating LQT3. Kis and Li (2024) [77] demonstrated the effectiveness of a monoclonal antibody (mAB-1) in normalizing the electrophysiological properties of iPSC-CMs. Using standard immunization techniques, they generated hybridoma cells from Balb/c mice spleen cells with high antibody titers, fused these with myeloma cells, and cultured them in HAT-supplemented RPMI 1640 medium. Six specific* KCNQ1*-targeting clones were selected and purified, with mAB-1 showing optimal function. In ATX-II-induced iPSC-CMs, mAB-1 effectively restored APD90 to baseline levels, suppressing EADs and arrhythmic activity. Together, these findings suggest that biological approaches have strong therapeutic potential in LQTS management.

## 5. Challenges and Prospects

### 5.1 Immaturity of Cells

iPSC-CMs often exhibit limited maturity, displaying embryonic-like electrophysiological characteristics. They lack the stability of adult cardiomyocytes, such as a stable action potential. Additionally, the absence of a T-tubule system restricts efficient intracellular calcium transfer, which impedes the analysis of calcium handling and triggered activity [78]. The resting membrane potential in iPSC-CMs is also less stable due to insufficient *I*_K1_ expression and an elevated “funny current” (If), affecting their ability to accurately replicate adult cardiomyocyte electrophysiology [79]. Furthermore, discrepancies in ion channel kinetics, such as those observed with *I*_Ks_, result in variable electrophysiological responses compared to mature heart cells. Several strategies have been explored to improve the maturity of iPSC-CMs. Gene transduction and current injection [80, 81], specifically through Kir2.1 transduction or simulated *I*_K1_ injection, enhance *I*_K1_ density, thereby promoting a more stable resting membrane potential and physiologically accurate AP morphology. Hormonal treatment, including thyroid hormone exposure, can induce features more similar to adult cardiomyocytes. Additionally, the culturing of cells on nano-structured or biomimetic substrates also supports structural and functional maturation by providing a more physiologically relevant environment. The optimization of differentiation conditions facilitates the generation of cells with predominantly atrial or ventricular characteristics, further increasing their specificity and maturity. Lastly, three-dimensional (3D) tissue culture [82, 83, 84] methods, such as microspheres or myocardial tissue strips, fosters stable beating and electrophysiological characteristics that closely resemble actual heart tissue. Collectively, these approaches enhance iPSC-CM maturity, improving their utility for electrophysiological and pharmacological screening studies.

### 5.2 Cellular Heterogeneity 

The heterogeneity of iPSC-CMs primarily arises from molecular variations during differentiation that are shaped by specific signaling pathways, growth factors, and cytokine responses. Differentiating cardiomyocyte subtypes, such as ventricular, atrial, and nodal cells, rely on precisely regulated signaling pathways such as the Wnt and BMP pathways. Even minor differences in the timing and strength of these signals can produce mixed populations with diverse phenotypes, thereby reducing iPSC-CMs homogeneity. For example, Wnt activates progenitor formation, whereas its inhibition promotes differentiation. BMP2 contributes to myocardial differentiation, while Notch and fibroblast growth factor (FGF) regulate cell fate and proliferation, ensuring coordinated cardiac development. Additionally, transcription factors such as NK2 homeobox 5 (NKX2.5), T-box transcription factor 5 (TBX5), and short stature homeobox 2 (SHOX2) play crucial roles in cell fate during differentiation. Variation in the expression of these factors leads to mixed ventricular and atrial phenotypes within the same culture, impacting the consistency of the generated iPSC-CMs. Culture conditions, whether two-dimensional (2D) or 3D, also affect cell-cell interactions and phenotypic stability.

To address these challenges, several strategies have been developed to increase the homogeneity of iPSC-CMs: (1) Optimization of the differentiation protocols through modulation of the Wnt and BMP pathways can lead to more specific iPSC-CM subtypes [85]. Small molecule inhibitors can be used to refine the timing and strength of pathway activation, resulting in more uniform cell populations. (2) Advanced co-culture systems with supportive cardiac cells, such as endothelial cells and fibroblasts, help to create a more physiological environment. This promotes stable differentiation and phenotypic consistency by mimicking natural cardiac signals. (3) Gene editing tools such as CRISPR/Cas9 can further improve iPSC-CM stability by reducing spontaneous gene expression changes, facilitating the development of more uniform cell lines suitable for disease modeling. (4) Defined culture media containing specific fatty acids and supplements that mimic the metabolic profile of the human heart contribute to more consistent maturation across iPSC-CMs populations. (5) Expanding iPSC-CMs in 3D bioreactors provides a controlled environment for dynamic cell growth and reduces heterogeneity compared to static cultures. Such bioreactor systems allow real-time adjustments of the growth conditions and support reproducible, large-scale differentiation. Standardization and scalability is essential to ensure that iPSC-CMs can be produced at the scale required for widespread clinical and research use. Lee *et al*. (2020) [86] recently developed a primordial 3D heart-like structure using mouse embryonic stem cells (ESCs) and FGF signaling. This structure included defined atrium, ventricle, pacemaker cells, smooth muscle cells (SMCs), and endothelial cells (ECs). Similarly, Hofbauer *et al*. (2021) [87] generated self-organizing “cardioids” using human iPSCs. These contained cells from the myocardium, epicardium, and endocardium, successfully mimicking the early development of heart chambers. However, integrating multicellular coupling models to study intercellular coupling properties and synchrony, and exploring the propagation of electrical signals and repolarization of iPSC-CMs in 3D tissue cultures or engineered heart tissues, remains a significant challenge.

### 5.3 Computational Models

Computational models are widely used to study mechanisms of cardiac automaticity and arrhythmias [88]. They offer precise control over parameters so that the compensatory effects seen in genetic animal models can be overcome, while also allowing simultaneous and detailed analysis of multiple components, including ion currents, membrane potentials, and intracellular concentrations. Consequently, these models have significant value in the CiPA initiative, which aims to predict the proarrhythmic risk of new drugs. However, the models are becoming increasingly complex as our understanding of cardiac electrophysiology deepens, since they integrate molecular ion channel dynamics, localized calcium handling changes, and post-translational regulation through signaling cascades [89].

Standard iPSC-CMs AP models typically encompass detailed dynamics of sodium (Na^+^), calcium (Ca^2+^), and potassium (K^+^) channels, which are used to study voltage changes under normal and pathological conditions. Many models are based on the AP models of mature cardiomyocytes, with parameter adjustments to accommodate the unique characteristics of iPSC-CMs. For example, the Paci model aims to replicate the dynamic characteristics of AP and calcium handling. This makes it suitable for simulating the effects of drugs on ion channels, and in particular for evaluating the proarrhythmic risk of new drugs. Additionally, this model can be adjusted to simulate different cardiomyocyte subtypes. However, iPSC-CMs often retain embryonic-like phenotypes, and hence the Paci model may not accurately reflect the structure and function of mature cardiomyocytes. Another model developed by Koivumäki *et al*. (2018) [90] focuses on the impact of the immature characteristics of iPSC-CMs on cellular function. It provides a detailed description of calcium handling and action potential properties, and offers a virtual platform for simulating disease mechanisms. This model is particularly suitable for studying differences in electrophysiological characteristics between iPSC-CMs and mature cardiomyocytes. It effectively simulates the impact of the lack of mature T-tubule structures in iPSC-CMs, allowing for an in-depth analysis of calcium handling abnormalities and their potential contribution to arrhythmias. It is useful for disease modeling and drug screening, helping to predict the specific effects of drugs on immature cardiomyocytes.

## 6. Conclusion

The application of iPSC technology in LQTS research represents a paradigm shift towards personalized medicine. iPSC-CMs provide unique insights into disease mechanisms, drug response, and genetic contributions, offering a robust platform for both basic and translational research. However, challenges remain in achieving full cardiomyocyte maturation and consistent cell differentiation. Continued advances in gene editing, 3D culture systems, and computational modeling will further bridge the gap between *in vitro* models and clinical practice, enhancing the predictive value of iPSC-derived models in LQTS research.

## Abbreviations

LQTS, Congenital Long QT Syndrome; iPSC, Human-Induced Pluripotent Stem Cells; iPSC-CMs, Human-Induced Pluripotent Stem Cell-Derived Cardiomyocytes; SCD, Sudden Cardiac Death; TdP, Torsade de Pointes; ICD, Implantable Cardioverter Defibrillator; LCSD, Left Cardiac Sympathetic Denervation; *I*_Ks_, Slow Delayed Rectifier Potassium Current; *I*_Kr_, Rapid Delayed Rectifier Potassium Current; *I*_Na,L_, Late Sodium Current; APC, Automated Patch Clamp; MEA, Multielectrode Array; APD, Action Potential Duration; FPD, Field Potential Duration; CDI, Calcium-Dependent Inactivation; WES, Whole Exome Sequencing; WGS, Whole Genome Sequencing; NGS, Next Generation Sequencing; VUS, Variants of Uncertain Significance; SNV, Single Nucleotide Variants; CiPA, Comprehensive in Vitro Proarrhythmia Assay; PBMBs, Peripheral Blood Mononuclear Cells; KI, Knock In; GM, Gene Modification; ATS, Andersen-Tawil Syndrome; JLNS, Jervell and Lange-Nielsen Syndrome; TM, Timothy Syndrome; TKOS, Triadin Knockout Syndrome; PPAR-δ, Peroxisome Proliferator-Activated Receptor Delta; BMP, Bone Morphogenetic Protein; FGF, Fibroblast Growth Factor; ESCs, Embryonic Stem Cells; SMCs, Smooth Muscle Cells; ECs, Endothelial Cells.
